# Dynamical controls on the longevity of a non-linear vortex : The case of the Lofoten Basin Eddy

**DOI:** 10.1038/s41598-019-49599-8

**Published:** 2019-09-17

**Authors:** Anthony Bosse, Ilker Fer, Jonathan M. Lilly, Henrik Søiland

**Affiliations:** 1 Geophysical Institute, University of Bergen and Bjerknes Center for Climate Research, Bergen, Norway; 2 Theiss Research, La Jolla, California USA; 3 Institute of Marine Research and Bjerknes Center for Climate Research, Bergen, Norway

**Keywords:** Physical oceanography, Fluid dynamics

## Abstract

The Lofoten Basin is the largest oceanic reservoir of heat in the Nordic Seas, and the site of important heat fluxes to the atmosphere. An intense permanent anticyclone in the basin impacts the regional hydrography, energetics, and ecosystem. Repeated sampling of this Lofoten Basin Eddy from dedicated cruises, autonomous profiling gliders, and acoustically-tracked subsurface floats enables the documentation of its dynamics and energetics over the course of 15 months. The eddy core, in nearly solid-body rotation, exhibits an unusually low vertical vorticity close to the local inertial frequency and important strain rates at the periphery. Subsurface floats as deep as 800 m are trapped within the core for their entire deployment duration (up to 15 months). The potential vorticity is reduced in the core by two orders of magnitude relative to the surroundings, creating a barrier. In the winter, this barrier weakens and lateral exchanges and heat flux between the eddy and the surroundings increase, apparently the result of dynamical instabilities and a possible eddy merger. Based on a simple energy budget, the dissipation timescale for the eddy energy is three years, during which wintertime convection seasonally modulates potential and kinetic energy.

## Introduction

Oceanic eddies — coherent swirling vortices of typically 10–100 km radius — are important conveyors of heat, salt, and energy, as well as biogeochemical and other tracers across the ocean^1–3^. Among various oceanic vortex structures, non-linear subsurface anticyclones with large Rossby number are known to be long-lived, cross oceanic basins, and disperse their properties slowly by mixing processes^4–8^. Characterized by a weakly stratified core below the thermocline, these eddies are relatively small and non-linear (i.e. with centripetal terms contributing non-negligibly in the momentum equation) compared to surface mesoscale^9^. Since the discovery of deep lenses of Mediterranean water in the Atlantic Ocean in the 1970s^10^, Submesoscale Coherent Vortices (SCV^11^) have been observed in convective basins (Greenland^12,13^, Labrador^7,14^ and Mediterranean^6,15^ Seas), in the Arctic Ocean^16,17^, as well as in subtropical oceans^4,18–21^. The generation of their weakly stratified cores often requires turbulent diapycnal mixing, believed to occur mainly either within the surface^22^ or bottom^23^ boundary layers. Most of the aforementioned observations have been opportunistic sampling by moorings, subsurface drifters, or more recently by autonomous profiling platforms (gliders, Argo floats, or ice-tethered profilers). Long-lived eddies are affected by the important seasonal changes that occur in their surroundings, such as enhanced submesoscale flows during winter^24–26^ and springtime restratification by mixed layer instabilities^27–31^. The longevity of SCVs is controlled by various factors such as their interactions with external flows, winter-intensified eddy activity, and deep vertical mixing in high-latitude environments. However, with some exceptions^4,8,32^, eddy life cycles remain largely undocumented, mainly due to the difficulty in detecting, following, and repeatedly surveying the same eddy over a seasonal cycle.

The Lofoten Basin Eddy (LBE) is an apparently permanent anticyclonic coherent eddy in the Nordic Seas, first observed in the 1970s^33,34^. The LBE is located in the deepest part of the Lofoten Basin, with a depth greater than 3000 m, close to 70°N and 3°E^35,36^. Its persistence was confirmed during the last decade by shipborne^35,37,38^ and glider^36^ measurements that captured its year-to-year coherence. The LBE is characterized by a 15–20 km radius, 1200 m thick core of Atlantic Water, swirling at intense velocities that reach 0.8 m s^−1^ at 600–800 m depth. The bathymetric depression in the center of the Lofoten Basin is hypothesized to attract warm anticyclones shed from the Norwegian Atlantic Slope Current^39,40^. Eddy mergers, as well as deep wintertime mixing, have both been suggested as important processes contributing to the longevity of the LBE^41–44^. However, the LBE has never been sampled at the high spatial and temporal resolution required to document its seasonal evolution and interaction with the environment.

**Figure 1:**
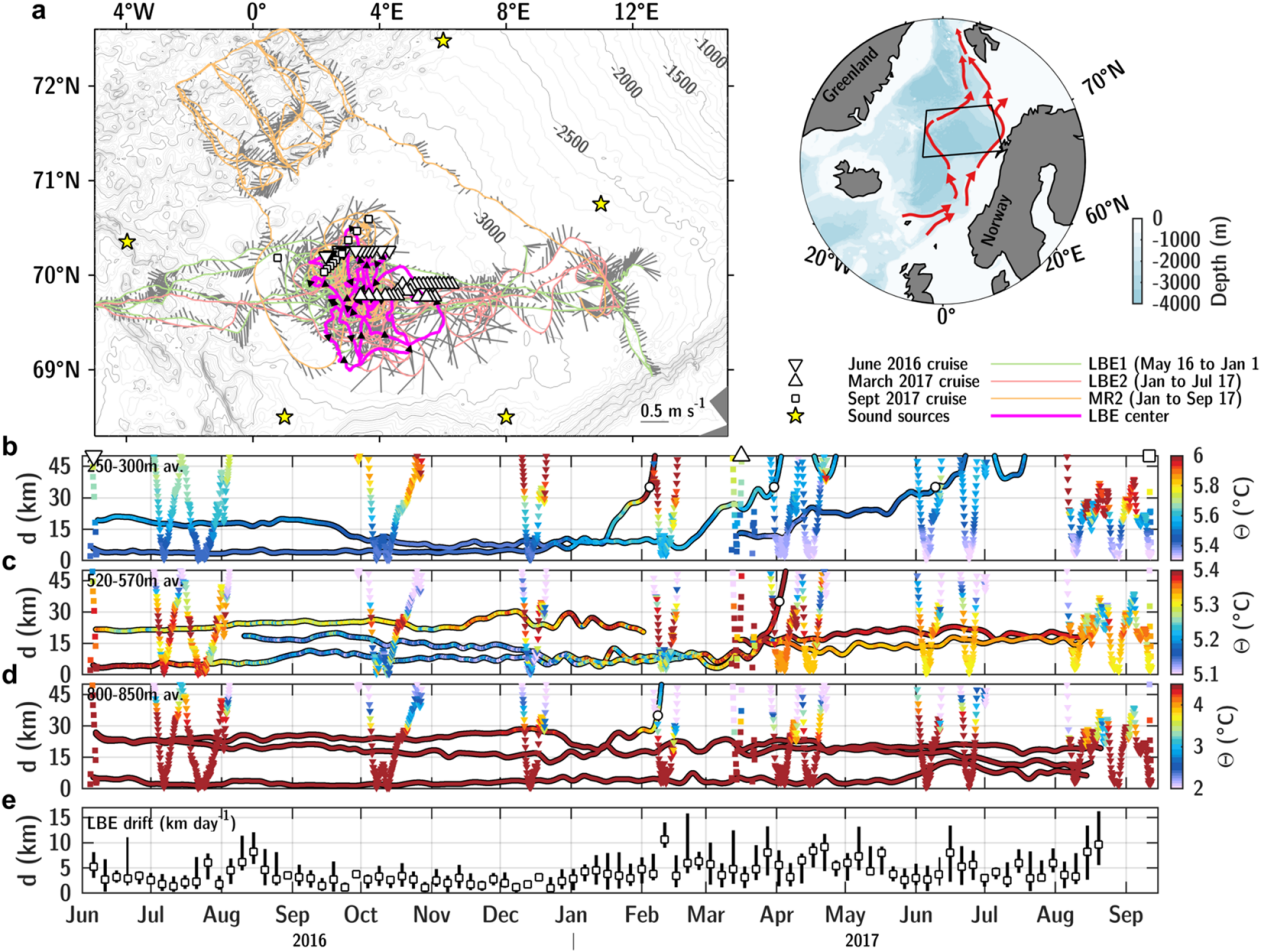
(**a**) Map of the PROVOLO fieldwork activities. White markers are CTD/L-ADCP carried out during the different cruises in the vicinity of the LBE: June 2016 (downward triangles), March 2017 (upward triangles) and September 2017 (squares). Colored lines show the Seagliders trajectories with depth-average current vectors in gray. The magenta line is the trajectory of the LBE center as inferred from subsurface RAFOS floats localized by sound sources (yellow stars). Bathymetry contours are drawn every 100 m. On the map of the Nordic Seas to the right, the study area is shown by the black box and red arrows represent the path of the two main poleward currents: the Norwegian Atlantic Frontal and Slope Currents. (**b**–**d**) Scatter plots of conservative temperature according to *d*, the distance to LBE center, at the three drifting depths of RAFOS floats: (**b**) 250–300 m; (**c**) 520–570 m; (**d**) 800–850 m. Glider (downward triangles) and cruise (squares) profiles are also drawn. White circles indicate the times when floats exit the LBE. (**e**) Bin-average drifting speed of LBE in 5-days intervals. Markers are median values, lines show 5^*th*^ and 95^*th*^ percentiles. Maps in panel (a) were generated using MATLAB R2018a (http://www.mathworks.com/).

Here, we describe the LBE dynamics using a unique set of observations collected from June 2016 to September 2017 in the framework of the PROVOLO project which aims to characterize water mass transformation processes and vortex dynamics in the Lofoten Basin. The experiment was designed to study the seasonal variability and interactions of the LBE with its environment. The data were collected from three research cruises, three Seaglider missions and eleven subsurface RAFOS floats (Fig. 1a). A detailed analysis of the heat budget of eddy core and radial structure of dynamical parameters reveals an important seasonal contrast in lateral exchanges with surroundings. Enhanced exchanges in winter are controlled by potential vorticity barrier and restratification processes at submesoscale. The role of merger events and winter convection in maintenance of the LBE, and implications for similar other coherent eddies are discussed.

## Results

### Eddy characteristics

**Figure 2:**
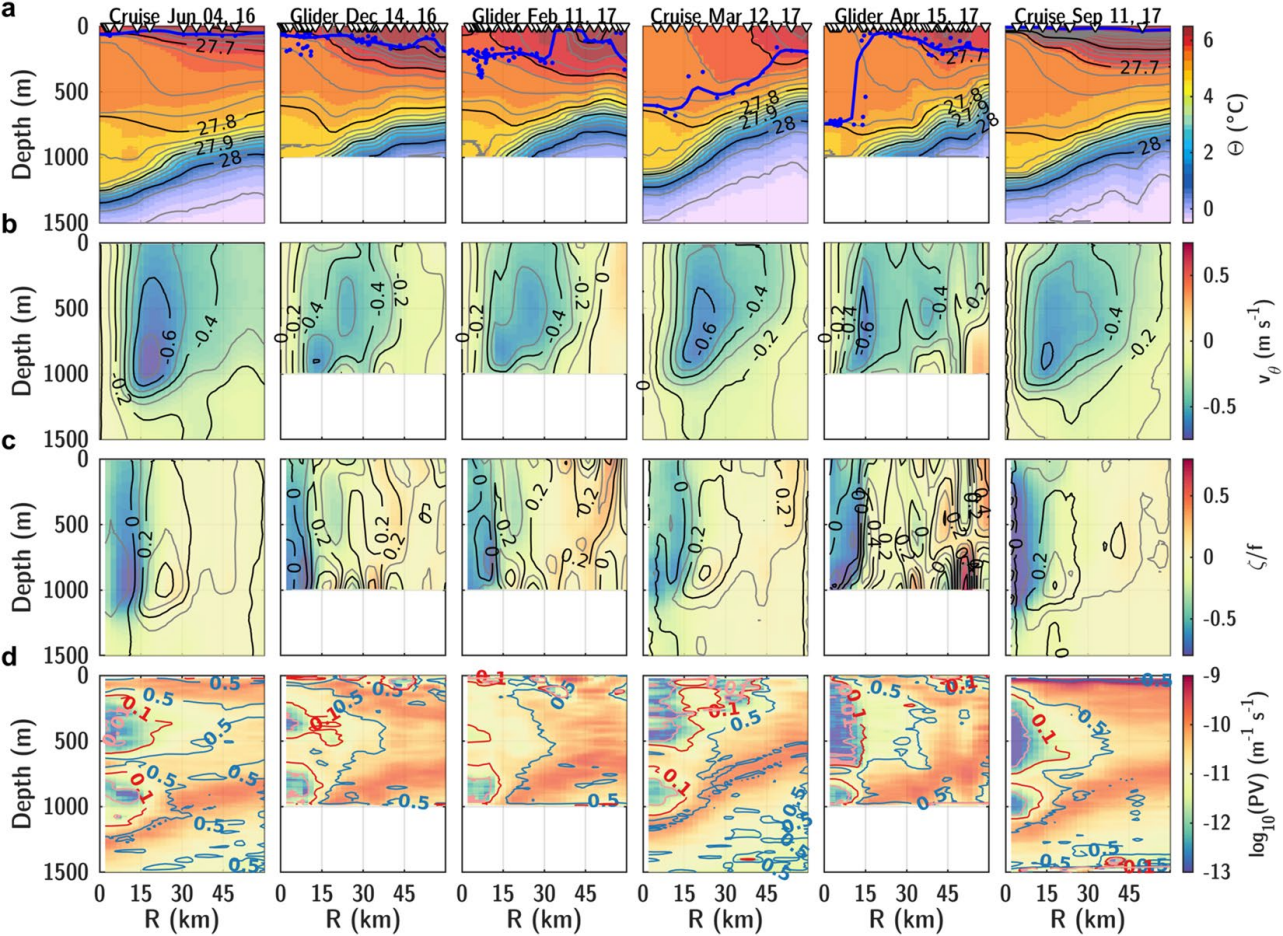
Seasonal evolution of the LBE as observed by gliders and ships. Cross-sections of (**a**) conservative temperature with contours of potential density anomalies, (**b**) azimuthal velocities, (**c**) relative vorticity with contours of strain rate normalized by *f*, (**d**) potential vorticity with contours of along isopycnal core to background PV ratio (light red corresponds to a reduction by a factor of 100, dark red of 10 and dark blue of 2). Blue dots are estimates of MLD from CTD profiles (blue line from optimally interpolated sections). White triangles in the upper panels show the position of cruise CTD/L-ADCP or glider profiles. Note that glider sections, due to their spiraling trajectories, did not capture the strain rate at the eddy periphery as accurately as synoptic shipborne sections.

The coordinated sampling described in Data and Methods allows a description of the LBE’s evolution during an entire seasonal cycle, see Fig. 2a. Deep mixed layers observed in the core reached 750 m by mid-April, deeper than the mixed layers typically observed in the basin^45^. A warm and fresh seasonal thermocline formed in the summer, as a result of solar heating and transport of fresh waters from the continental shelf, with the upper part of the LBE remaining colder than its surroundings. Modified Atlantic Water, characterized by temperatures higher than 4 °C, was seen to extend down to 1200 m in the LBE core, about 500 m deeper than its surroundings in the Lofoten Basin. Radial sections — four from ship-based observations and 23 from gliders — characterize the LBE as having a core radius of 15.0 ± 2.4 km and a peak azimuthal velocity of −68.4 ± 6.3 cm s^−1^ at the depth of 860 ± 80 m, in accordance with previous estimates^35–38^. The velocity structure is found to resemble that of a non-isolated weakly shielded vortex^46^ (similar to the well-known Rankine vortex structure, see Supplementary Fig. S5) characterized by an outward slow decrease in azimuthal velocities to about 30 cm s^−1^ at 30 km from the eddy center, and to 20 cm s^−1^ at 45 km. The mean Rossby number of the eddy (as defined in the Data and Methods section) was −0.68 ± 0.13, and the minimum core vorticity was −0.87 ± 0.12* f*, confirming the crucial importance of the non-linear centrifugal force in the LBE balance^36,47^ and its marginal stability regarding inertial instability^48^. The RAFOS floats trapped in the LBE reveal the temporal evolution of its drifting speed, see Fig. 1d. During the 15 months of RAFOS floats deployment, the LBE center traveled along a 1850 km long track at a speed of 1 to 5 km day^−1^, with episodic peaks reaching 10–15 km day^−1^. The average eddy displacement speed roughly doubled in the winter of 2017, from 3.0 ± 0.3 km day^−1^ before January 2017 to 6.1 ± 0.4 km day^−1^ from February to April. Whilst topographic *β*-effect can explain the general down-slope counter-clockwise movement of the LBE around the deepest part of the basin^49,50^ (Fig. 1a), the LBE drift is also influenced by interaction due to neighboring eddies. As the deformation radius (*R*_*d*_ proportional to the stratification) decreases in winter, so does the contribution from *β*-effects (proportional to $\beta {R}_{d}^{2}$^51^). Thus, the increase in the LBE displacement speed could be due to the evolution of its environment, in particular to the wintertime energizing of the background eddy field^24–26^. More frequent interactions with cyclones forming transient dipoles could episodically speed up the LBE translation^52,53^.

### Eddy Lagrangian coherence

**Figure 3:**
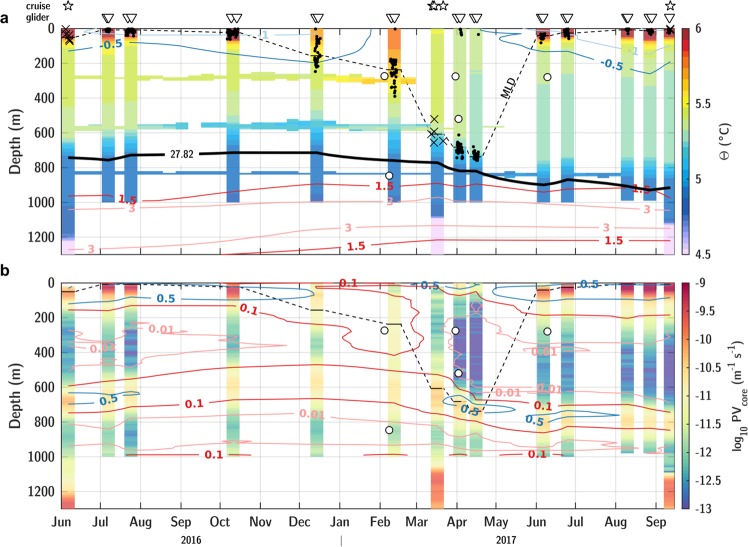
(**a**) LBE core temperature binned in 10 days and 10 m intervals from June 2016 to September 2017. The thin dashed line follows the median MLD observed in each interval. Individual MLD observations from shipborne CTD (resp. gliders) profiles are marked by black crosses (resp. dots). The thick line shows the 27.82 isopycnal used to estimate the heat storage rate. The white circles show the depth and time when RAFOS floats exited the LBE. Above the panel, white stars (resp. triangles) show the time of each LBE radial characterization by cruises (resp. gliders). Blue and red contours represent temperature difference relative to the background. (**b**) Same for the LBE core PV with blue and red contours representing the core to background PV ratio.

Lagrangian floats deployed inside the LBE within 30 km of its center stayed trapped for more than one year, testifying to its remarkable coherence, see Fig. 1b–d. Notably, the fraction of floats exiting the LBE decreased with increasing depth. All three floats deployed at 250–300 m depth were ejected after three to ten months. In contrast, three out of four floats deployed at 500–550 m and at 800–850 m remained trapped for their entire deployment period, see Table 1. Lateral exchanges between the eddy and its surroundings seem more active near the surface, where the eddy currents are weaker than at depth. While there are relatively few data points, the ejection of floats was observed to occur preferentially during winter, specifically during two brief time intervals in early February and April when floats at two depths were ejected simultaneously, see the white circles in Fig. 3a. The estimated distance between the float and the eddy center, inferred as described in the Data and Methods section, increased suddenly whenever a float crossed the radial distance threshold of about 30 km, despite relatively strong velocities of about 0.3 m s^−1^ at that distance, see Fig. 2b. It is worth noting that three additional RAFOS floats deployed at 40 km from the eddy center at 250, 550 and 800 m, and not otherwise described here, were ejected rapidly after only a couple of revolutions. The eddy vorticity was roughly constant from the center up to about half of eddy radius, a consequence of inner core in approximately solid-body rotation^35,36^, and with an implied strain rate associated with the azimuthal velocity of zero. However, beyond the velocity maximum and to about 30 km from the center, the inferred strain rate increased to a maximum value of 0.2–0.4 *f* (Fig. 2c and Supplementary Fig. S5), with the outer boundary of the region of enhanced strain rates coinciding with the outer boundary of the trapping region as inferred from the Lagrangian floats. On dynamical grounds, one expects eddies approaching the LBE with a vorticity similar to or smaller than the strong strain rate found at the LBE periphery to be sheared apart^54,55^, surrendering their heat and salt content in the form of filaments in the rim region around the LBE core^56,57^.

**Table 1:** RAFOS floats characteristics deployed at less than 30 km from the LBE center (serial number, average drifting depth, initial distance to the eddy center, date of deployment, date of LBE exit, and residence time within the LBE).

ID	Mean Depth (m)	*r*_0_ (km)	Deployment date	LBE exit	*T*_*LBE*_ (days)
1293	287	5.66	Jun 06, 16	Mar 30, 17	298
1283	298	13.2	Mar 16, 17	Jun 09, 17	85
1304	247	19.3	Jun 08, 16	Feb 04, 17	241
1285	561	4.34	Jun 06, 16	—	435
1266	509	16.6	Mar 16, 17	Apr 02, 17	17
1261	567	18.6	Aug 12, 16	—	370
1258	586	21.6	Jun 08, 16	—	239
1448	830	5.37	Jun 06, 16	—	435
1265	855	17.4	Mar 16, 17	—	158
1203	814	24.8	Jun 08, 16	—	435
1211	795	25.7	Jun 08, 16	Feb 08, 17	245

### Heat budget of the LBE core

**Figure 4:**
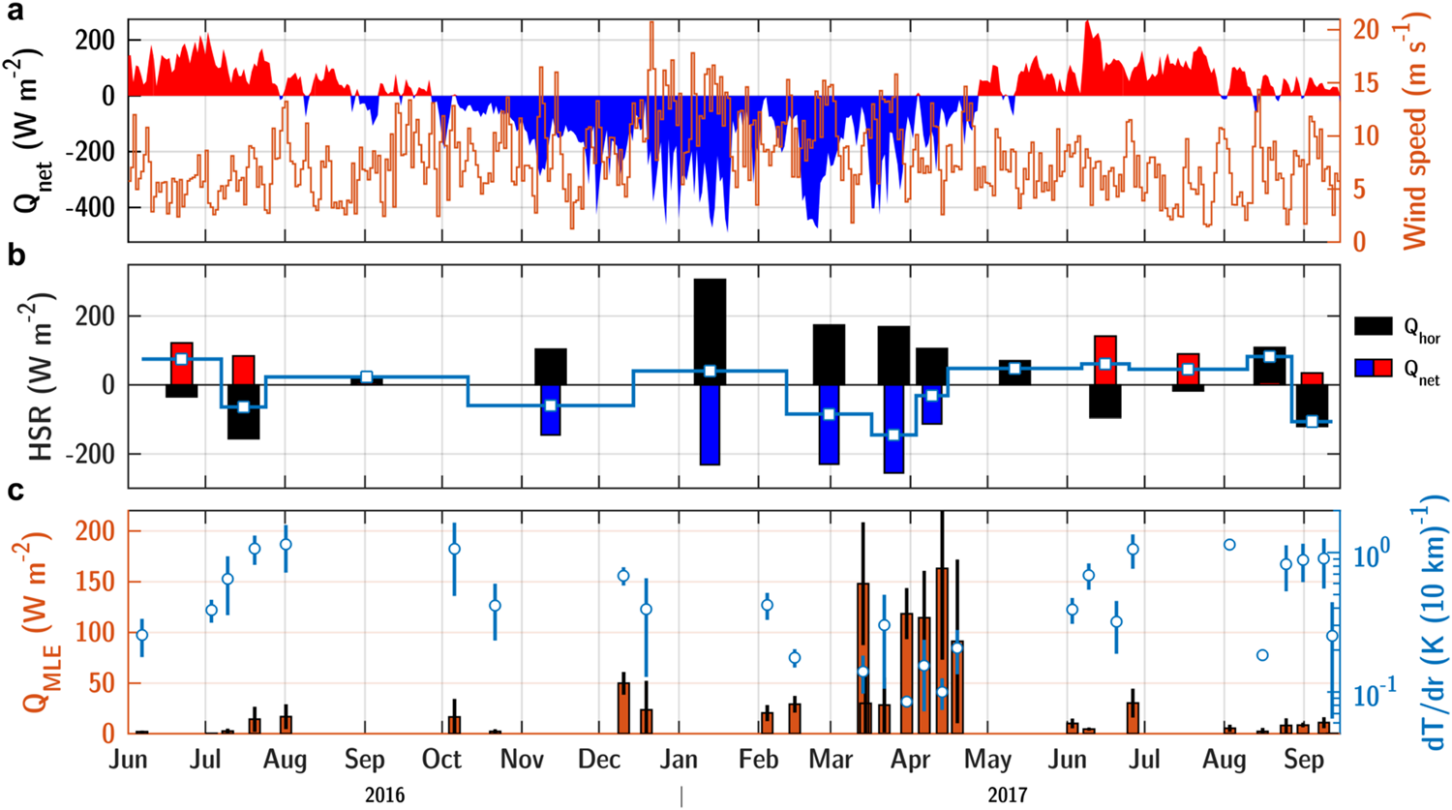
Time series of (**a**) daily-average net heat fluxes (left axis) and wind speed (right axis) from ERA-Interim reanalysis at 70°N and 3°E, (**b**) heat storage rate of the LBE core (thick blue line) with surface net heat flux (red and blue bars) and horizontal heat convergence (black bars), (**c**) heat flux by mixed layer eddies *Q*_*MLE*_ (left axis) and radial temperature gradient average of the MLD (right axis) at the outskirt of the LBE. The vertical error bars are mean values with one standard deviation observed in the core/rim frontal region (i.e., at ±5 km around the velocity maximum).

Over a seasonal cycle, the LBE heat storage rate shows important variability, especially during winter, see Fig. 4. Between two consecutive cross-sections in December and February, at the apex of winter characterized by an average surface net heat loss of ${Q}_{net}=-\,230\,{\rm{W}}\,{{\rm{m}}}^{-2}$, the LBE core is surprisingly estimated to gain heat at a rate of about 50 W m^−2^; the temperature of the upper 400 m increases by 0.16 °C during this interval, see Fig. 2a. During this period of positive heat storage rate, the compensating lateral heat flux is equivalent to a surface flux of +310 W m^−2^. This heat flux integrated over the area of the mean LBE radius represents about 10^18^ J. The upper part of the LBE is always characterized by a cold anomaly of 0.5–1 °C, see Fig. 3a. The warm waters of the LBE rim provides a heat source for the warming of the core observed during the winter. The heat transport into the Lofoten Basin, and eventually into the LBE, has been hypothesized to be sustained by mesoscale eddies shed from the Norwegian Atlantic Slope Current^58,59^. Exploring this hypothesis, one finds that the heat convergence into the LBE core observed between December and February is equivalent to the heat contained in a 250 m thick mesoscale eddy with a typical +1 °C anomaly and radius of 17 km^60^, suggesting eddy merger as a reasonable mechanism to account for the observed warming of the LBE.

### Potential vorticity barrier

**Figure 5:**
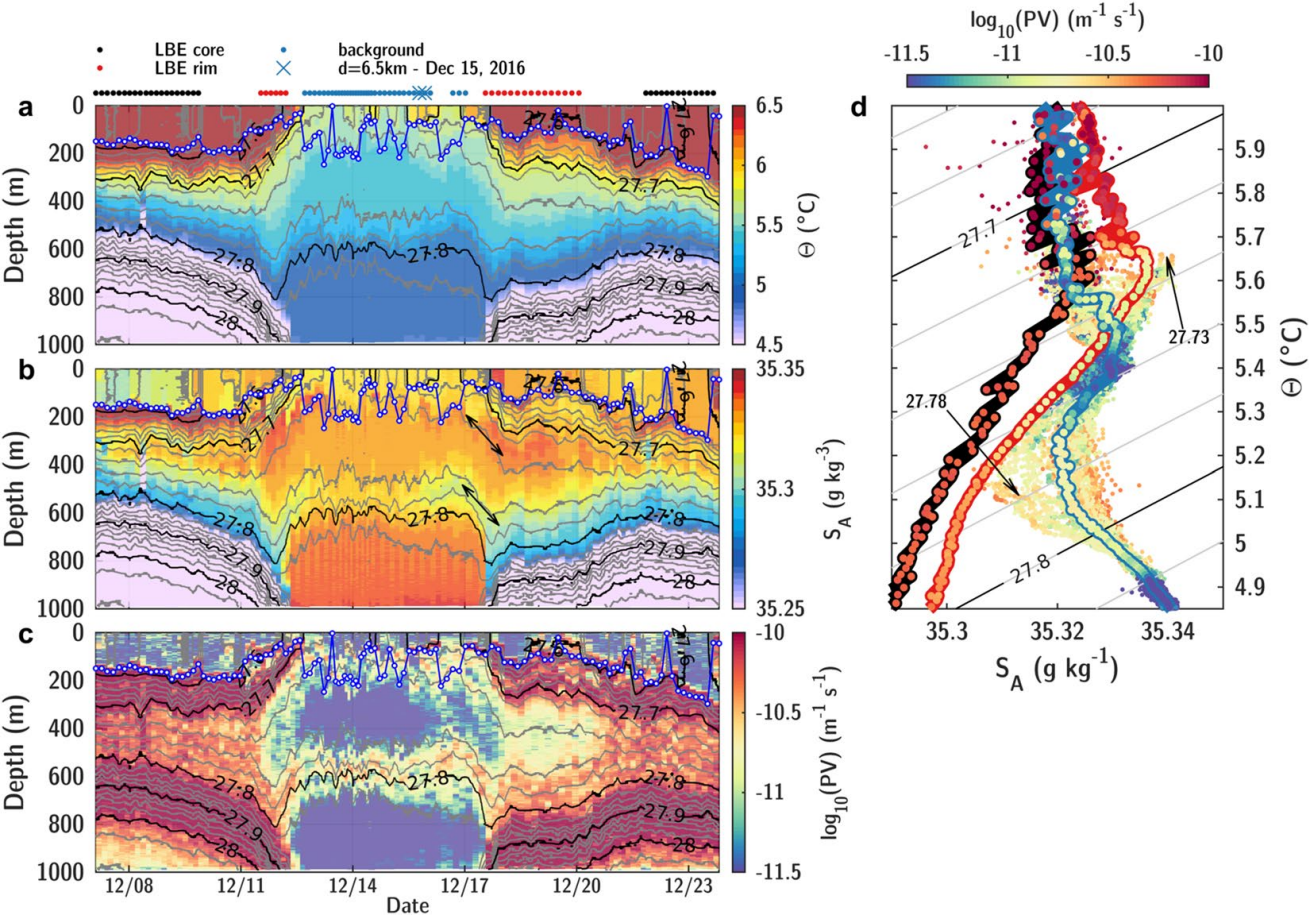
Along-track glider sections of (**a**) conservative temperature, (**b**) absolute salinity, (**c**) potential vorticity during two LBE cross-sections performed in December. To reconstruct along-track PV, stratification was computed from individual density profiles, whereas vorticity and radial buoyancy gradients were interpolated according to depth and radial distance from the corresponding optimally interpolated cross-sections. Above the panels, dots are colored according to defined LBE regions (core, rim and background, see Data and Methods). The blue line with white dots indicates MLD. (**d**) $\Theta /{S}_{A}$ diagram of core profiles colored according to their PV. The thick blue (resp. red and black) line shows the mean profile in the core (resp. rim and background) averaged along isopycnals with colored dots indicating PV averaged along isopycnals.

In absence of forcing and dissipation, potential vorticity (PV) is a Lagrangian tracer of the adiabatic along-isopycnal circulation in the ocean interior^61^. PV can thus be used to diagnose the ease of lateral exchanges at the eddy outskirt below the mixed layer. The strong anticyclonic vorticity and weakly stratified layers in the LBE core reduced the PV by two orders of magnitude when compared along isopycnals with a background at rest, see Figs 2d and 3b. The eddy PV structure was drastically modified over the seasonal cycle by deep vertical mixing in winter. The PV signature of the deep core from 800 to 1100 m was present year-round, while the upper core PV contrast weakened from December to March (Fig. 3b). As the mixed layer deepened, a newly-formed weakly stratified core in the upper LBE restored the strong PV gradients of two orders of magnitude. Dynamical constraints governed by the core/background PV gradient thus suggest that lateral exchanges with the surroundings are favored in early winter in the upper part of the eddy, when heat is also observed to converge into the LBE core (Fig. 3a). In December, prior to the intense warming period, PV gradients in the upper 500 m between the core and the background weakened. The reduced dynamical barrier facilitated the intrusion of warm waters from the eddy rim, where the upper 250 m was on average 0.9 °C warmer (Fig. 3a). From mid-December to mid-February, including days with intense cooling reaching 300–400 W m^−2^, the PV below the mixed layer increased (Fig. 3b). This is contrary to the expected destruction of PV during this period of continuous destabilizing surface buoyancy fluxes, and implies that the stratified waters required to increase the PV of the eddy core must come from its periphery. This is confirmed by the along-track glider measurements, see Fig. 5c.

### Seasonal restratification at submesoscale

The LBE core was characterized by mixed layers deeper than 600 m from mid-March to mid-April, whereas shallower mixed layer depths were observed in the LBE rim and farther outside due to earlier spring restratification (Fig. 2a and Supplementary Fig. S2). These deep mixed layers were separated by a steep density front, driven by temperature difference between the core and rim regions. The core/rim temperature front within the mixed layer was approximately 1 °C per 10 km during stratified conditions, and decreased by one order of magnitude whilst mixed layer deepened, see Fig. 4c. The potential energy of the front can form submesoscale eddies by mixed layer baroclinic instabilities^27,28^ transferring buoyancy (i.e., heat) to the less buoyant side of the front (the LBE core, here). An important variability in MLD and upper temperature and salinity was observed along the glider tracks across the LBE core, see Fig. 5a,b. This variability occurred abruptly over 1–2 glider dives, corresponding to 5–10 km horizontally. In December, baroclinic instability of a surface mixed layer front has a typical growth rate of 1 day (~$3N/\Lambda f$^28^ using $f=1.4\,{.10}^{-4}\,{{\rm{s}}}^{-1}$ the local inertial frequency, $\Lambda \sim 5\,{.10}^{-4}\,{{\rm{s}}}^{-1}$ the geostrophic shear and $N\sim 1.7\,{.10}^{-3}\,{{\rm{s}}}^{-1}$ the buoyancy frequency) and length scale of 5 km (~4*Nh*/*f*  ^28^ using a mixed layer depth of *h* = 100 m).

In the $\Theta /{S}_{A}$ space, the signature of waters originating from the rim region was observed propagating into the core (fresher water along the 27.78 isopycnal, saltier along 27.73, see Fig. 5b,d). This signal was also associated with an increase in PV (i.e., restratification), and locally reduced the MLD (Fig. 5c). Two months later, the T-S properties of the core and rim regions transformed by winter convection were isopycnally homogeneous, and only differed by the presence of warmer waters at the surface in the rim (see Supplementary Fig. S4), thus indicating the importance of lateral mixing within the mixed layer. By mid-March to mid-April, when the MLD was the deepest, mixed layer eddies could contribute to restratification by a substantial heat flux of 100–150 W m^−2^ (Fig. 4c), comparable to the heat convergence deduced from the heat storage rate from February to April (Fig. 3c). From December to February, however, this flux was only 10–50 W m^−2^, well below the magnitude of the reported lateral heat convergence. Hence, other mechanisms must be considered to close the LBE heat budget (e.g., merger events, lateral fluxes by dynamical instabilities and filaments). The lateral exchange of heat/salt across the LBE core showed an important seasonality. Restratification mechanisms were more active during winter, when the mixed layer was growing and horizontal PV gradients were weakening.

### Interactions with external flows

**Figure 6:**
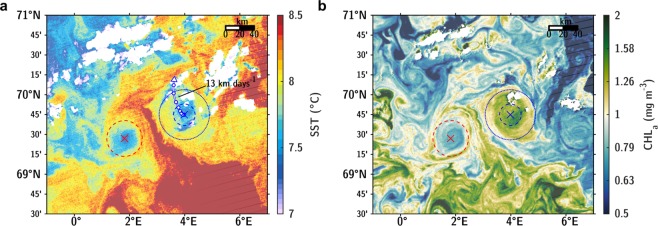
Sea surface (**a**) temperature and (**b**) chlorophyll-a concentration on June 9, 2017 measured by MODIS-Aqua (cloud-masked L2 map at 1-km high-resolution). The white line in (**a**) shows the LBE trajectory inferred from RAFOS floats during the week following the satellite observation with dots indicating daily position. The maximum translation speed of 13 km day^−1^ on June 14 is annotated. Blue circles are drawn at 15 and 35 km radial distance from the eddy center, representing approximately the position of the velocity maximum and of the outer rim region of the LBE. A red circle with 25-km radius is drawn at the approximate location of the nearby cyclone.

Five RAFOS floats were ejected from the LBE at the beginning of February, April and at mid-June. During the last event, a cloud-free satellite image of sea surface temperature and chlorophyll-a of the LBE revealed a nearby cyclone of similar scale (Fig. 6). Submesoscale features are ubiquitous with a warm filament located between the LBE and the closeby cyclone, as well as numerous eddies of O(10-km) scale unresolved by our measurements. A few days later, the LBE translation speed increased rapidly with a daily average of 13 km day^−1^ (peaking at 18 km day^−1^) and a RAFOS float at 250 m was ejected. Self-advection of asymmetric dipoles follows a curved path bending toward the dominating vortex^53^. The curved path of the LBE trajectory confirms its interaction with a weaker cyclone, see Fig. 6a. At translation speeds of 15–20 cm s^−1^, parts of the LBE periphery and surface with comparable azimuthal velocities can lose their Lagrangian coherence. Five to six peaks in the LBE drift of similar amplitude can be spotted during the survey period, see Fig. 1e. Importantly, RAFOS floats appear to be ejected during such phases. Accelerations in the LBE drift, resulting from potential interactions with cyclonic eddies, could thus be an important mechanism for the exchange of peripheral waters.

The RAFOS floats trapped in the LBE core showed constant vorticity over long periods. During a merger (e.g., with a surface mesoscale anticyclone with non-zero barotropic component), a decrease in vorticity (in absolute value) is expected by lateral mixing of vorticity as the interaction takes place, especially in the upper layer. The deeper layer could experience vortex squishing reinforcing the anticyclonic vorticity by PV conservation. But during vortex merger, the conservation of PV may not hold anymore^56^. A decrease in vorticity was observed in the upper and deeper layers in January, following the important heat convergence, suggesting a merger event could have occurred, see Supplementary Fig. S7. Outside the winter period, the high vorticity of the core in solid-body rotation was marginally affected, especially in the deep core, during possible interactions with external flows. It is worth noting that the deep core below 800 m also became thinner (Fig. 3a), without being affected by winter mixing. So, a loss of mass has probably occurred. This is supported by the ejection of a RAFOS float drifting at 800 m in February. The energy cascade toward small scale processes (eddies, filaments, instabilities) in presence of a deep mixed layer is likely responsible for the disruption of the regular oscillatory motions of the RAFOS floats with high frequency perturbations aliased by the 6-h sampling rate, see Supplementary animation. Once spring restratification has started, geostrophic adjustment resets the LBE into a new state of stable vorticity (see Fig. 2 and Supplementary Fig. S6).

### Energy budget

**Figure 7:**
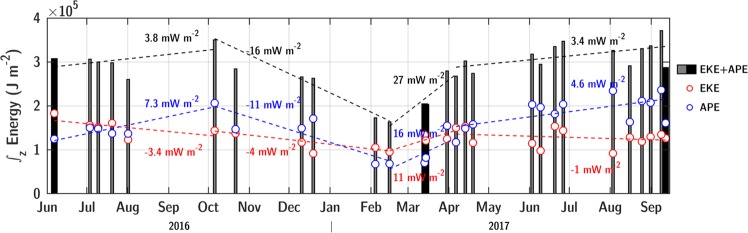
Time series of vertically integrated EKE (red), APE (blue) and total energy (bars) per surface unit estimated from the LBE radial sections. Gray bars are estimates from glider sections and black bars from ship sections. Trends during selected seasonal periods are indicated.

The sum of eddy kinetic energy (EKE) and available potential energy (APE) in the LBE slowly increased from April to October, but showed an important seasonal signature with a decrease by a factor two from October to February followed by a strong increase from February to April (Fig. 7). These variations were mainly driven by the seasonal cycle of APE, strongly affected by the difference in timing and depth reached by winter convection in the LBE and the surroundings from October to June (Fig. 2a). The restratification of the background began early, from mid-February (see Supplementary Figs S2), while the LBE core experienced deep mixing until mid-April. This timing offset caused the APE to strongly increase in the spring-summer transition, see Supplementary Fig. S8. Over the same period, the LBE was energized with a steep increase in EKE at a rate of +11 mW m^−2^. The rest of the year, EKE monotonically decreased at a rate of −1 to −4 mW m^−2^. In the energy seasonal cycle, APE and EKE were restored to pre-winter values when MLD reached maximum values in April, stressing the importance of winter deep convection in maintaining the LBE energetics. Note that energy levels differed between summer 2016 and 2017, illustrating that the seasonal cycle reported here was observed during a particular seasonal cycle, but may change from year to year.

Energy sinks of the LBE can be due to turbulent dissipation rates driven by wind, internal mixing processes and bottom drag. Considering the yearly average wind speed of 8 m s^−1^ in the region, an average ocean surface velocity of 0.3 m s^−1^ and near-bottom currents of 0.1 m s^−1^ (measured by L-ADCP, see Data and Methods), estimates of the sinks by the surface and bottom drags are *S*_*w*_ = 1.0 mW m^−2^ and *S*_*b*_ = 2.6 mW m^−2^, respectively. In June 2016, an average dissipation rate by turbulent mixing of about 0.5 mW m^−2^ was reported from microstructure observations^38^. Seasonal fluctuations in dissipation rates are expected, but remain to be quantified. On average, friction due to winds and bottom currents summed up with turbulent mixing can thus explain the decrease in EKE observed outside the winter period. At this rate, and assuming that APE would eventually be drained after conversion into EKE, the LBE would dissipate in about three years. Note that instability of the vortex itself might be effective at dissipating the LBE. Such processes were found to develop slowly on timescale of several months^44^ and cannot be quantified using the present data set.

## Discussion

The interaction of an apparently permanent, subsurface, non-linear anticyclone in the Lofoten Basin with its environment is documented for the first time by multiple *in situ* surveys over a full seasonal cycle. New aspects of the Lofoten Basin Eddy’s seasonality, depth dependence and exchange with its surroundings are documented. Earlier numerical^42–44^ and remote sensing^62^ studies emphasized the importance of eddy merger in the LBE dynamics, with 1–4 merger events occurring every year^47^. A numerical model with horizontal resolution of 4 km was able to successfully reproduce a LBE with the observed radius^43,44,47^, but underestimated the maximum azimuthal velocity and core vorticity by a factor of two^47^. The  core vertical vorticity close to −*f*, strong strain rate at the eddy periphery as well as the seasonally intensifying potential vorticity barrier are expected to impose dynamical controls on the interaction of the LBE with external eddies. Such interactions would likely be characterized by small scales and submesoscale dynamics, which would only be fully resolved by higher-resolution models.

Between December and February, the core LBE heat content was observed to increase by the amount contained in a typical mesoscale anticyclone, contemporaneously with the restratification of the upper core, thus providing indirect but compelling evidence of an eddy merger event. Even with the dense *in situ* sampling of the LBE, with 27 radial cross-sections over 16 months, merger events remain challenging to capture and document. Such mergers could account for a significant part of the heat and salt budgets, whereas these calculations indicate the LBE energy evolution seems instead to be governed by wintertime mixing. It should be noted that our observations are from one particular year, while modeling studies have reported interannual variability in the LBE^43,44^. Several years of LBE observations are therefore required to firmly establish the seasonal evolution of the LBE energy and dynamics. It appears submesoscale mixed layer eddies could be important in lateral exchange across the density front encompassing the eddy core. Considering the dissipation of EKE through internal mixing, bottom friction and wind drag, and ignoring surface heat losses, we estimated that a decay timescale of approximately three years allowed wintertime vertical mixing to refuel the eddy during this particular year. Given this long decay timescale, interannual variability in wintertime mixing as well as changes in basin properties and stratification (e.g., the recent freshening of the Atlantic Water^45,63^) could also affect the LBE evolution and survival. High resolution realistic numerical models resolving the frontal submesoscale processes could be used to delve into such questions and guide future fieldwork.

Finally, the Lagrangian coherence of Submesoscale Coherent Vortices and deep vertical mixing generate important localized vertical fluxes of nutrients, organic matter and carbon, with biogeochemical and biological responses^13,64,65^. The importance of these aspects for the LBE deserves further attention.

## Data and Methods

### Cruise data

Observations in the LBE were made during the three PROVOLO cruises from *RV Håkon Mosby* in June 2016 and from *RV Kristine Bonnevie* in March 2017 and September 2017. Conductivity–Temperature–Depth (CTD) profiles were acquired using a Sea-Bird Scientific SBE 911plus system, with pressure, temperature, and salinity data accurate to respectively ±0.5 dbar, ±0.002 °C, and ±0.003 g kg^−1^. The CTD data were processed using the SBE software following the recommended procedures. Salinity samples were analyzed in order to correct, when necessary, CTD-derived values. Conservative Temperature $\Theta$, Absolute Salinity *S*_*A*_ and potential density anomaly ${\sigma }_{\Theta }$ were calculated using the TEOS-10 Gibbs Seawater Oceanographic toolbox^66^. Profiles of horizontal currents were acquired by a pair of 300-kHz lowered acoustic Doppler current profilers (L-ADCP) attached to the CTD rosette, operated in master-slave mode with 8-m vertical bins. The L-ADCP data were processed using the velocity inversion method^67^ implemented in the LDEO software version IX-12^68^, with typical horizontal velocity uncertainties of 2–3 cm s^−1^. All current measurements were corrected for the magnetic declination.

In the field, the LBE was identified using a vessel-mounted ADCP. Several CTD/L-ADCP sections from the LBE center to about 80–100 km radius were carried out. A total of 15, 30, and 13 CTD/L-ADCP profiles were taken respectively in the vicinity of the LBE during the June 2016, March, September 2017 cruises. One radial section was performed during both the June 2016 and September 2017 cruises, while two radial sections were performed during the March 2017 cruise.

### RAFOS floats

RAFOS floats are subsurface drifters tracked by acoustic sound sources^69^; the acronym is the reverse of SOFAR or SOund Fixing And Ranging. The trajectories of eleven RAFOS floats deployed during the June 2016 and March 2017 cruises at three different depths and less than 25 km from the LBE center are analyzed here, see Table 1. They were programmed to surface in August 2017, except for float 1258 that was programmed to surface at the beginning of February 2017. In addition to acoustic travel times, from which the float position was calculated based on triangulation from two or more sound sources every six hours, the floats also measured pressure and temperature. Using a wavelet-based method^70,71^, individual Lagrangian trajectories were decomposed into an oscillatory portion, representing the rotational currents in the moving frame of the eddy, together with a residual representing the motion of the eddy center. As individual residual trajectories have some variability, a mean residual trajectory was obtained from the five floats that remained the longest in the LBE (i.e., floats 1285, 1211, 1203, 1448, and 1265). From this Lagrangian data, the LBE center position can be relatively precisely estimated from June 2016 to August 2017, with a mean standard error of 0.56 km. A float was considered to exit the LBE when its distance to center exceeded 35 km. Remarkably, six floats out of eleven, generally those from the deepest deployments and initially closer to center, remained in the LBE during their whole deployment period (up to 15 months).

### Seaglider missions

Gliders are autonomous instruments that collect data along a sawtooth trajectory between the surface and a maximum depth of 1000 m, travelling at a speed of 10–30 km per day^72^. Between two successive dives, one may estimate the depth-averaged currents (DAC) by comparing the glider dead-reckoning positions to actual GPS fixes. The particular type of gliders we used, Seagliders^73^, are equipped with a navigation mode suited to sample intense mesoscale eddies rotating faster than the glider displacement through the water, and which has been successfully used during past glider missions visiting the LBE^36^. By adapting their heading relative to the on-board estimated DAC, the gliders can move inward and outward within eddies, leading to spiraling trajectories. Our Seagliders were first guided to the approximate position of LBE, near 70°N and 3°E, with help of seal level anomaly maps from satellite altimetry produced and distributed by Copernicus Marine Environment Monitoring Service. When caught by intense currents, the LBE core could be identified by Atlantic Water extending beyond 1000 m.

From June 2016 to September 2017, a pair of autonomous Seagliders occupied the Lofoten Basin, as part of the PROVOLO project, with a turnover to a new pair of gliders in January 2017. These four missions were all successful and lasted for 6 to 8 months. Three of these four missions visiting the LBE have been used here (LBE1, LBE2 and MR2, see Fig. 1a). The Seagliders measured the conductivity, temperature and depth using a Sea-Bird SBE37 unpumped CTD system. The typical vertical velocity of the gliders was 8 cm s^−1^ with a sampling rate of 10–20 s, resulting in a vertical resolution of 0.8–1.6 m. Salinity was calculated after applying a thermal lag correction^74^. Finally, assuming the glider CTD measurements were less accurate than the shipborne ones, glider temperature and salinity profiles were offset in order to match the LBE core T-S properties measured by calibrated shipborne CTD casts performed within a 30 day window.

### Atmospheric reanalysis

Atmospheric forcing were obtained from ECMWF’s ERA-Interim reanalysis^75^ over the time period from June 2016 to September 2017. Surface net fluxes *Q*_*net*_ were computed from net shortwave and longwave contributions, as well as latent and sensible heat fluxes, as ${Q}_{net}={Q}_{sw}+{Q}_{lw}+{Q}_{sens}+{Q}_{lat}$, with downward heat fluxes defined to be positive. Time series of fluxes were extracted at 70°N and 3°E, corresponding to the mean position of the LBE. These were computed by differentiating cumulated fields saved every 3 h and initialized at 0 and 12 h. Winds were retrieved from reanalysis fields at 0, 6, 12 and 18 h.

### Reconstruction of radial cross-sections

#### Temperature, salinity and velocity fields

A radial cross-section of the LBE was constructed for each ship-based or glider section into or out of the eddy core. To do this, a cylindrical coordinate system moving with the LBE was defined using the eddy center deduced by RAFOS floats from June 2016 to August 2017. For the September 2017 cruise and the last three glider cross-sections in August 2017, when RAFOS floats  had all completed their mission, the LBE center was instead inferred using L-ADCP velocities^38^ or glider DAC^36,76^. The LBE is assumed to be circular, and radial cross-sections of temperature, salinity, and azimuthal velocities were then constructed from shipborne CTD/L-ADCP profiles using an optimal interpolation method. This method used the two-dimensional Gaussian correlation function given by

1 $${cov}(R,Z)=E\delta (R,Z)+(1-E)\,{e}^{-{R}^{2}/{L}_{r}^{2}-{Z}^{2}/{L}_{z}^{2}}$$

with $\delta (R,Z)$ being the two-dimensional Dirac delta function, $E=0.05$ the relative error, the radial scale *L*_*r*_ set to a typical LBE radius ${L}_{r}\equiv 15\,{\rm{km}}$, and the vertical scale *L*_z_ to a typical seasonal thermocline thickness ${L}_{z}\equiv 15\,{\rm{m}}$. For the glider cross-sections, temperature and salinity measurements were objectively interpolated using the same procedure as for shipborne measurements. The azimuthal component of the DAC was decomposed into geostrophic and cyclostrophic components following ref.^15^, and the cyclogeostrophic balance was resolved. Each inward or outward glider spiral capturing the velocity maximum of the LBE core provided a characterization of the LBE, for 23 glider sections in total, see Fig. 3 and Supplementary Fig. S3.

For each LBE cross-section, the eddy radius *R*_*m*_ was defined to be the radius at which the maximum azimuthal velocity occurred. The Rossby number is then defined as $Ro\equiv 2{V}_{m}/{R}_{m}f$ with *V*_*m*_ the peak velocity and *f* the Coriolis parameter. The LBE core was identified as ship or glider profiles reaching to 10 km or less from the eddy center, while rim region was defined as those extending between 15 and 35 km and having average 900–1000 m temperatures between 1.5 and 4.5 °C, see Supplementary Fig. S1.

#### Potential vorticity and strain rate

The Ertel’s Potential Vorticity (PV, with units of m^−1^ s^−1^) is defined as^61^

2 $$PV\equiv \frac{(\nabla \times v+f\hat{z})\cdot \nabla b}{g}$$

where *v* is the three-dimensional velocity, *g* is the gravitational acceleration, $b\equiv -\,g\rho /{\rho }_{0}$ is the buoyancy with $\rho$ and ${\rho }_{0}$ being the density and reference density, respectively, and $\hat{z}$ is the vertical unit vector. For a steady circular eddy having an azimuthal velocity of *v*_*θ*_, one finds

3 $$PV(r,z)=\frac{1}{g}({N}^{2}(f+\zeta )-\frac{\partial {v}_{\theta }}{\partial z}\frac{\partial b}{\partial r})$$

with $\zeta ={r}^{-1}{\partial }_{r}(r{v}_{\theta })$ being the relative vorticity, and ${N}^{2}\equiv {\partial }_{z}b$ the buoyancy frequency. PV was computed from the estimated azimuthal velocity and potential density from each LBE cross-section. The LBE PV field was then compared along isopycnals to the PV of a motionless background fluid, given by $f{N}_{out}^{2}/g$, with *N*_*out*_ being the average profile of stratification between 60 and 100 km from the eddy center and within ±15 days around each LBE cross-section; this was extended to 30 days in the cases when no profiles matched the 15 day criterion. In order to mitigate the influence of any surrounding of mesoscale eddies, a criterion was additionally applied to the average 900–1000 m temperature (see Supplementary Fig. S1). The strain associated with the LBE’s azimuthal velocities under the assumption of circularity is given by $\eta (r,z)\equiv {\partial }_{r}{v}_{\theta }-{r}^{-1}{v}_{\theta }$. For a circular flow, the magnitude of this strain rate also quantifies the deviation from solid-body rotation, for which $\eta$ would vanish.

### Heat budget and mixed layer eddies

The one-dimensional heat storage rate (*HSR*) within the LBE core is evaluated, following ref.^77^, by computing the temporal difference of the 10-day averaged heat content integrated from the surface down to the 27.82 isopycnal, a density level not reached by wintertime mixing (Fig. 3a). We make a standard assumption by neglecting the vertical heat flux due to diapycnal mixing at the base of the reference layer. However, changes in the depth of the reference isopycnal can affect the apparent heat content (assuming no changes in eddy radius), so the variation in the layer thickness *h* and vertical velocity were retained. The heat storage rate is thus defined as^77^

4 $$HSR(t)\equiv {\rho }_{0}{c}_{p}h\frac{\partial {T}_{a}}{\partial t}=-\,({T}_{a}-{T}_{-h})\,(\frac{\partial h}{\partial t}+{w}_{-h})+{Q}_{hor}+{Q}_{net}$$

where ${\rho }_{0}$ = 1028 kg m^−3^ is a reference density of seawater, *c*_*p*_ is the specific heat of seawater, *T*_*a*_ is the layer average temperature, and *T*_−*h*_ and *w*_−*h*_ are the temperature and vertical velocities at the layer base of thickness *h*, respectively, *Q*_*hor*_ represents the equivalent lateral heat flux required to balance the LBE heat budget given the surface net heat flux, *Q*_*net*_ (given by the ERA-Interim atmospheric reanalysis). Mean upward vertical velocities of 10^−5^ m s^−1^ were considered to be representative for the base of the LBE core^47^. The equation 4 was then solved for *Q*_*hor*_ between consecutive LBE cross-sections.

The restratification of baroclinic fronts by mixed layer eddies can be parametrized and expressed as an equivalent surface heat flux of ${Q}_{MLE}={\rho }_{0}{c}_{p}{C}_{e}{({\partial }_{r}b)}^{2}{H}^{2}/g{\alpha }_{T}\,f$^28^, with ${\rho }_{0}$ again being the density of seawater, *c*_*p*_ the specific heat capacity of seawater, g the gravitational acceleration, *α*_*T*_ the thermal expansion coefficient of seawater, ${\partial }_{r}b$ the radial buoyancy gradient, and *H* the mixed layer depth. Mixed layer was computed from density profiles using a 0.03 kg m^−3^ threshold^78^. ${C}_{e}=0.06$ is a nondimensional coefficient determined empirically from numerical studies^28^.

### Energy budget

The estimated Eddy Kinetic Energy (EKE) and Available Potential Energy (APE) per unit surface area in the LBE were calculated by integrating the following expression radially from the LBE center to 1.5 times the core radius (R_m_), and vertically from the surface down to 1500 m for shipborne cross-sections and to 1000 m for glider cross-sections 

5 $$EKE=\frac{1}{\pi {(1.5{R}_{m})}^{2}}\,{\int }_{r}\,2\pi rdr\,{\int }_{z}\,dz[\frac{1}{2}{\rho }_{0}{v}_{\theta }{(r,z)}^{2}]$$

6 $$APE=\frac{1}{\pi {(1.5{R}_{m})}^{2}}\,{\int }_{r}\,2\pi rdr\,{\int }_{z}\,dz[\frac{1}{2}{\rho }_{0}{N}_{out}^{2}(z)\xi {(r,z)}^{2}]$$

with ${\rho }_{0}$ being the density of seawater, *v*_*θ*_ the azimuthal veloities, $\xi$ the isopycnal displacement relative to the background, whose stratification is given by *N*_*out*_^79^. As the LBE extends below 1000 m, part of the LBE energy is not captured by gliders. The ratio of the energy integrated down to 1500 m to that integrated down to 1000 m is evaluated from the four ship sections. Those ratios are found to be 1.12 ± 0.04 for EKE and 1.34 ± 0.09 for APE; note that the variability about the mean is small in each case. Glider estimates integrated to 1000 m were then multiplied by those ratios to represent the vertically integrated EKE or APE per unit surface area of the LBE. Energy contained deeper than 1500 m is estimated to be negligible: a 1500 m thick bottom layer rotating at a mean barotropic velocity of 0.1 m s^−1^ would increase EKE by only 3%, while stratification as well as isopycnal displacement are small below this depth.

Kinetic energy sinks due to surface stress is estimated as ${S}_{w}={\tau }_{{\bf{w}}}\cdot {{\bf{v}}}_{{\bf{s}}{\bf{u}}{\bf{r}}{\bf{f}}}$ with ${\tau }_{{\bf{w}}}={\rho }_{a}{C}_{d}^{a}|{v}_{w}-{v}_{surf}|({{\bf{v}}}_{{\bf{w}}}-{{\bf{v}}}_{{\bf{s}}{\bf{u}}{\bf{r}}{\bf{f}}})$ with ${\rho }_{a}=1.2\,{\rm{kg}}\,{{\rm{m}}}^{-3}$ the density of air, ${C}_{d}^{a}=0.0012$ the surface drag coefficient for moderate winds^80^, **v**_**w**_ the wind vector and **v**_**surf**_ the surface ocean velocity vector. Considering a homogeneous wind field over the eddy and with winds parallel to but exceeding surface ocean velocity, we have ${S}_{w}\sim -\,{\rho }_{a}{C}_{d}^{a}|{v}_{w}|{v}_{surf}^{2}$. At the ocean bottom, the energy sink becomes ${S}_{b}={\tau }_{{\bf{b}}}\cdot {{\bf{v}}}_{{\bf{b}}{\bf{o}}{\bf{t}}}=-\,{\rho }_{0}{C}_{d}^{o}|{v}_{bot}{|}^{3}$ with ${\rho }_{0}$ the density of seawater, ${C}_{d}^{o}=0.0025$ the bottom drag coefficient^81^, and **v**_**bot**_ the bottom ocean velocity vector.

## Supplementary information

RAFOS floats, gliders and ship trajectories.

Supplementary material

## Data Availability

Data collected during the PROVOLO project are available at the Norwegian Marine Data Center (Cruise data: 10.21335/NMDC-1093031037, Seaglider data: 10.21335/NMDC-980686647). Seaglider data were processed using a toolbox developed by Bastien Queste at the University of East Anglia (https://bitbucket.org/bastienqueste/uea-seaglider-toolbox.git). ERA-Interim reanalysis can be freely downloaded for research purposes from the ECMWF’s website (https://www.ecmwf.int). Level-2 satellite data from MODIS-Aqua are made freely-available by NASA Goddard Space Flight Center (Ocean Color, 10.5067/AQUA/MODIS/L2/OC/2018; Sea Surface Temperature, 10.5067/AQUA/MODIS/L2/SST/2014). Lagrangian analysis tools developed by Jonathan Lilly are contained in a freely-available Matlab toolbox (jlab: A data analysis package for Matlab, v. 1.6.6, http://www.jmlilly.net/jmlsoft.html).
