# Supplementary material for: Dynamical controls on the longevity of a non-linear vortex : The case of the Lofoten Basin Eddy
Source: Sci Rep. 2019 Sep 17;9:13448. doi: 10.1038/s41598-019-49599-8 (PMC6748989; doi:10.1038/s41598-019-49599-8)
Supplement: Supplementary file 2 — Supplementary material [file 41598_2019_49599_MOESM2_ESM.pdf]

Supplementary informations to "Dynamical controls on the longevity of a non-linear vortex: The case of the Lofoten Basin Eddy"

A. Bosse[1], I. Fer[1], J. M. Lilly[2], H. Søiland[3]

[1]Geophysical Institute, University of Bergen and Bjerknes Center for Climate Research, Bergen, Norway

[2]Theiss Research, La Jolla, California, USA

[3]Institute of Marine Research and Bjerknes Center for Climate Research, Bergen, Norway

anthony.bosse@uib.no

July 10, 2019

1. Illustration of LBE tracking and the accomplished sampling

An animation illustrates the displacement of the Lofoten Basin Eddy (LBE) from the 1st of June 2016 to 15th of September 2017 with the trajectories of the eleven RAFOS floats used in the study, as well as the ships and Seagliders tracks. Triangles show shipborne CTD profiles and a 30 km radius circle centered at the LBE is drawn as reference.

2. Core/rim/background profiles selection

The criteria used to discriminate profiles in the LBE and outside were defined following Bosse et al. [2018] (Figure S1). The core showed very little temperature variability at depth between 900 to 1000 m (4.89±0.01°C). All profiles in the core were selected at less than 10 km (about two thirds of the LBE radius). As the eddy rim (defined between 15 and 35 km, i.e. between one to two radii) contained more variability from interactions with surrounding flows, an additional criterion on deep temperature ($1.5°C < \Theta_{900-1000} < 4.5°C$) was applied in order to remove cold profiles taken outside the eddy. Finally, the background region was chosen from 60 to 100 km (corresponding to four to six radii) with an additional temperature criterion ($0°C < \Theta_{900-1000} < 0.6°C$) used to discard mesoscale features generating cold/warm deep temperature anomalies. This was especially important when calculating the Available Potential Energy of the LBE, as it could be biased if the background stratification is affected by a surrounding

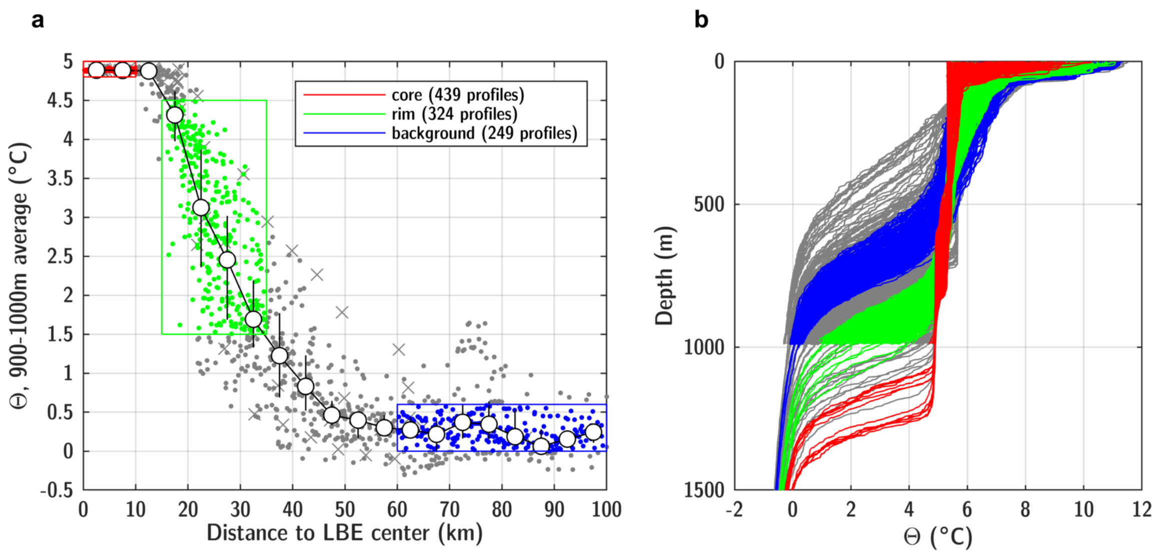

Figure S1: *(a) Average temperature in 900-1000 m according to the distance to the LBE center. Individual points are glider (dots) and ship (crosses) profiles. A colored box corresponds to the criteria used to discriminate profiles in the core, rim and background regions. (b) Vertical temperature profiles with colors corresponding to each regions.*

eddy. In the end, several hundreds of profiles satisfied each region, enabling to look at their seasonal evolution.

3. Seasonal evolution of the rim and background regions

The seasonal evolution of the water column properties in the rim and background regions differed from the LBE core. Especially the timing and depth of the deepest mixed layer varied quite substantially: about 600 m is reached by the end of March in the rim (Figure S2-a), unlike only 400 m by mid-February in the background (Figure S2-b). For comparison, the deepest MLD of approximately 800 m in the LBE core was reached by mid-April. Like the LBE core, the rim region was weakly stratified and characterized by a cold anomaly at surface compared to the background.

4. Inventory of ship and glider LBE radial sections

In order to provide to the reader a complete view of the LBE sampling during the study period (May 2016 to September 2017), the 4 ship and 23 glider radial sections are shown

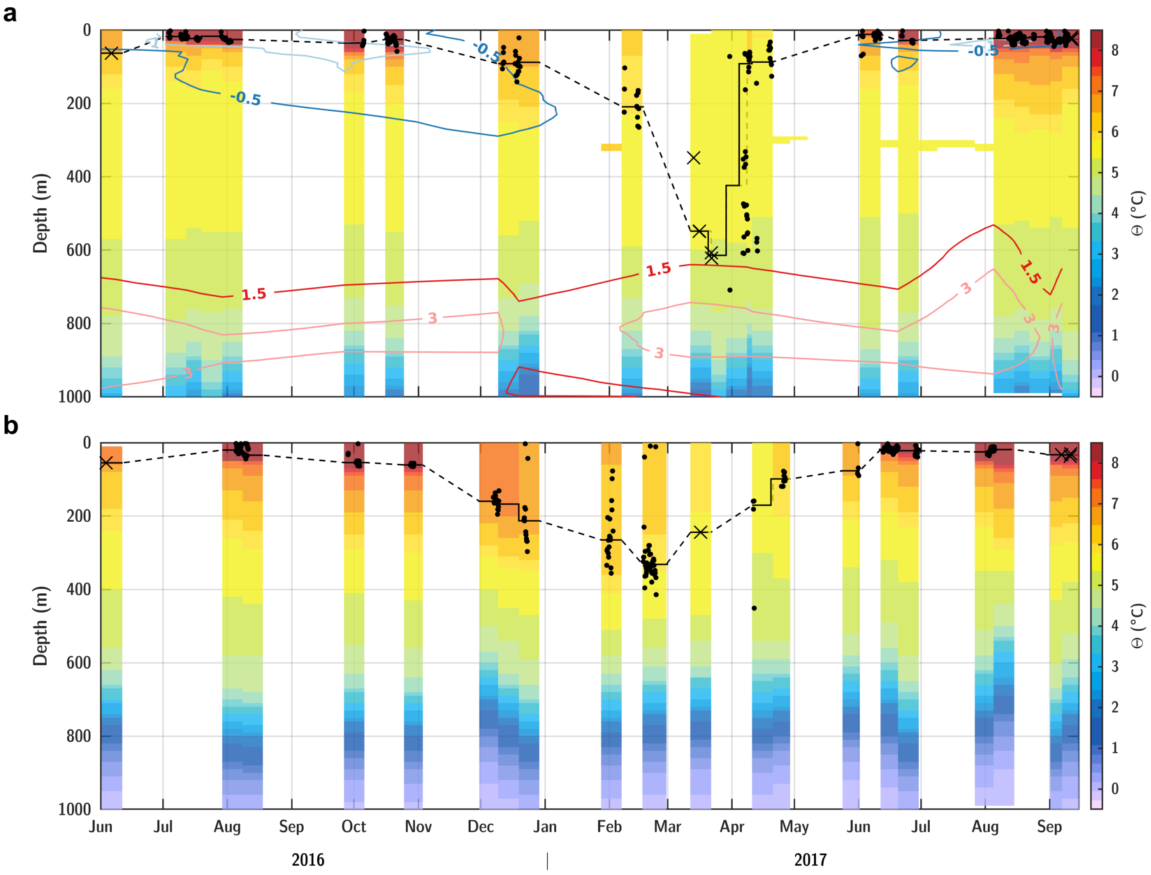

Figure S2: *(a) Depth-time evolution of conservative temperature in the rim region. Contours show temperature difference with the background. Black dots are mixed layer estimates from glider (dots) and ship (crosses) profiles. The black line follows the median mixed layer depth in 10-day intervals. (b) Same for the background region.*

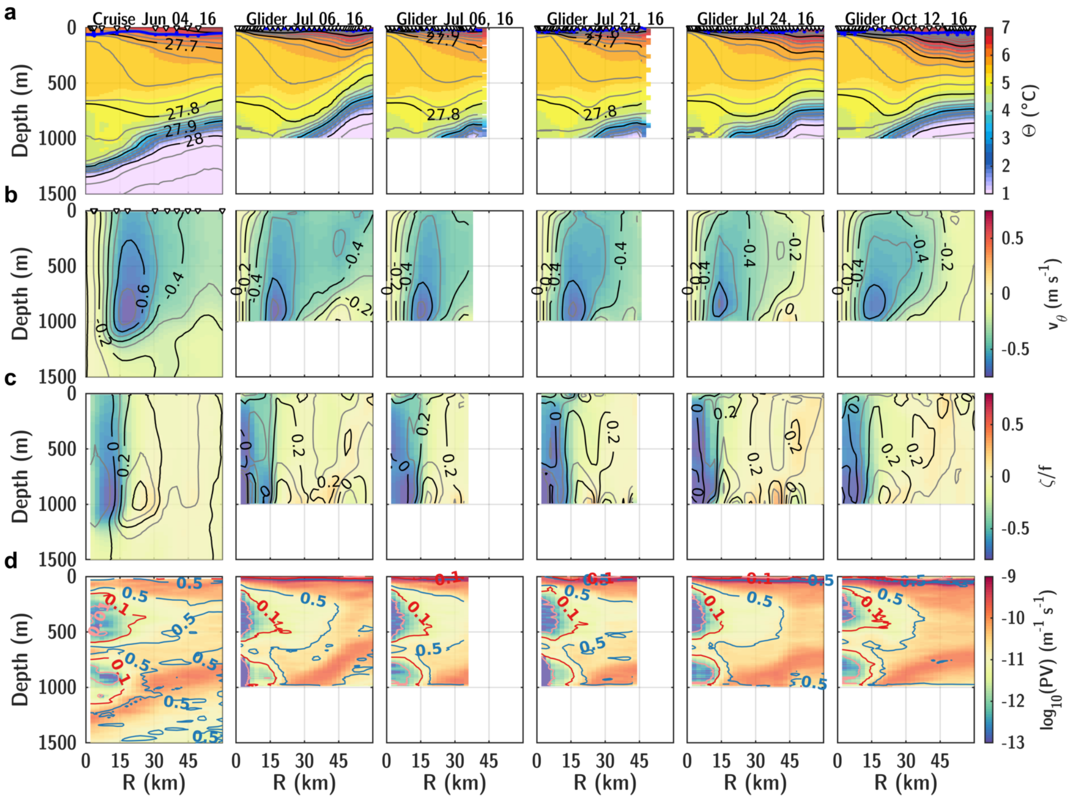

Figure S3: *LBE radial sections in chronological order with: (a) temperature with contours of potential density anomalies; (b) orbital velocities; (c) relative vorticity with contours of strain rate normalized by f; (d) potential vorticity with contours of along isopycnal core to background PV ratio (light red corresponds to a reduction of factor 100, dark red of factor 10 and dark blue of factor 2). Blue dots are estimates of MLD from CTD profiles (blue line from optimally interpolated sections). White triangles in the upper panels show the position of cruise CTD/L-ADCP or glider profiles. Figure continues 1/3.*

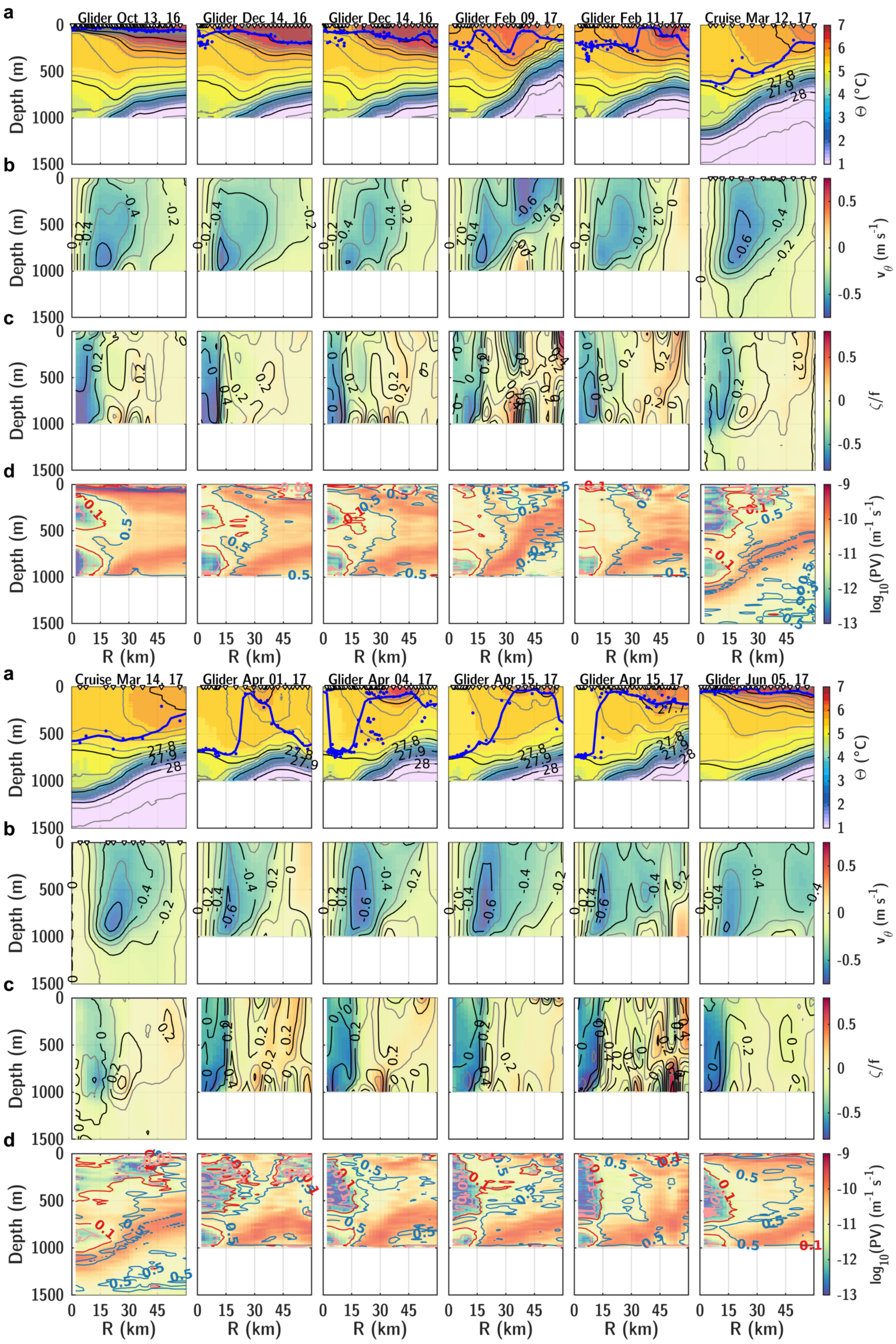

Figure S3: *Figure continuation, 2/3.*

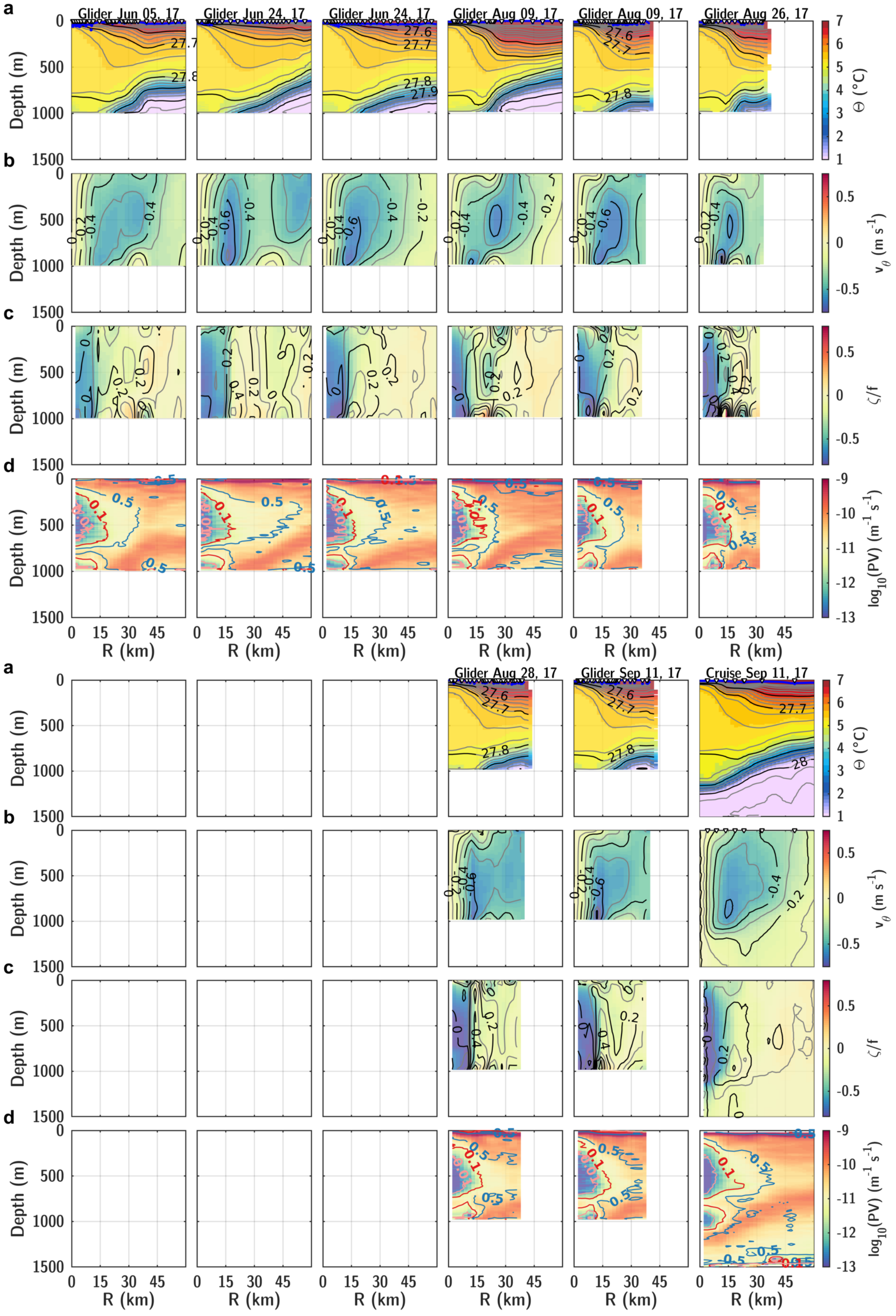

Figure S3: *Figure continuation, 3/3.*

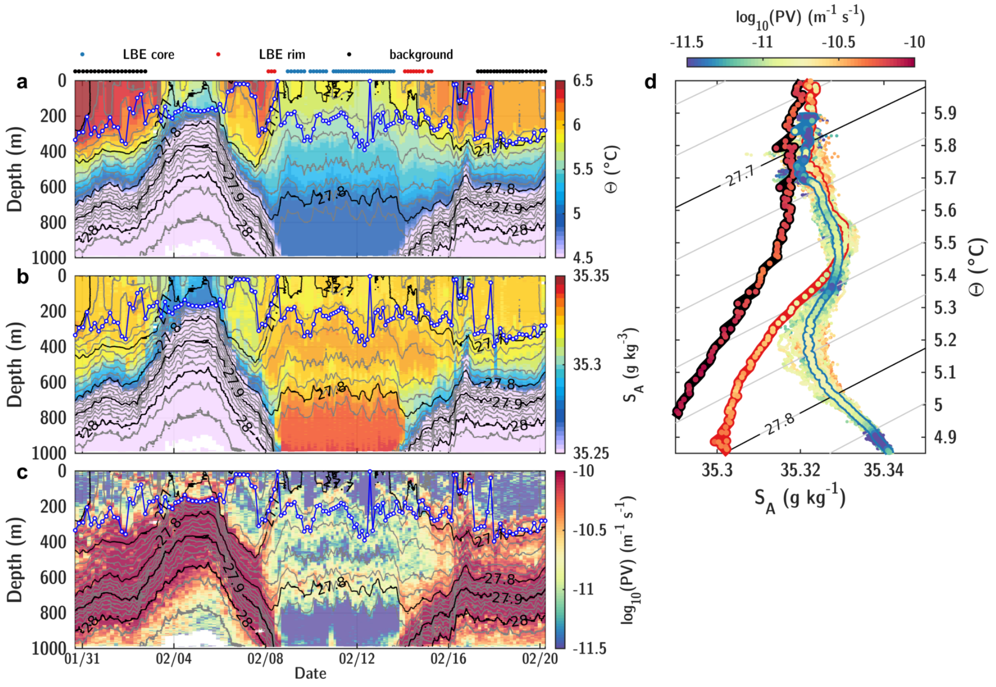

Figure S4: *Along-track glider sections of: (a) conservative temperature; (b) absolute salinity; (c) potential vorticity during two LBE samplings performed at the beginning of February. To reconstruct along-track PV, stratification was computed from individual density profiles, whereas vorticity and radial buoyancy gradients were interpolated from the corresponding radial sections. Above the panels, dots are colored according to pre-defined LBE regions (core, rim, background). The blue line with white dots indicates MLD. (d) T-S diagram of core profiles colored according to their PV. The thick blue (resp. red and black) line shows the mean profile in the core (resp. rim and background) averaged along isopycnals with colored dots indicating PV averaged along isopycnals. Figure continues 1/3.*

in chronological order in Figure S3.

5. Winter homogenization and restratification of the core

The history of the lateral mixing between the core and the rim of the LBE during winter is illustrated by samplings of the LBE at three different stages : the deepening of winter mixing in February (Figure S4), the apex of deep mixing in the LBE core with restratification already started in the rim/background regions in April, and stratified conditions in the LBE in June. This complements the section presented in the manuscript in De-

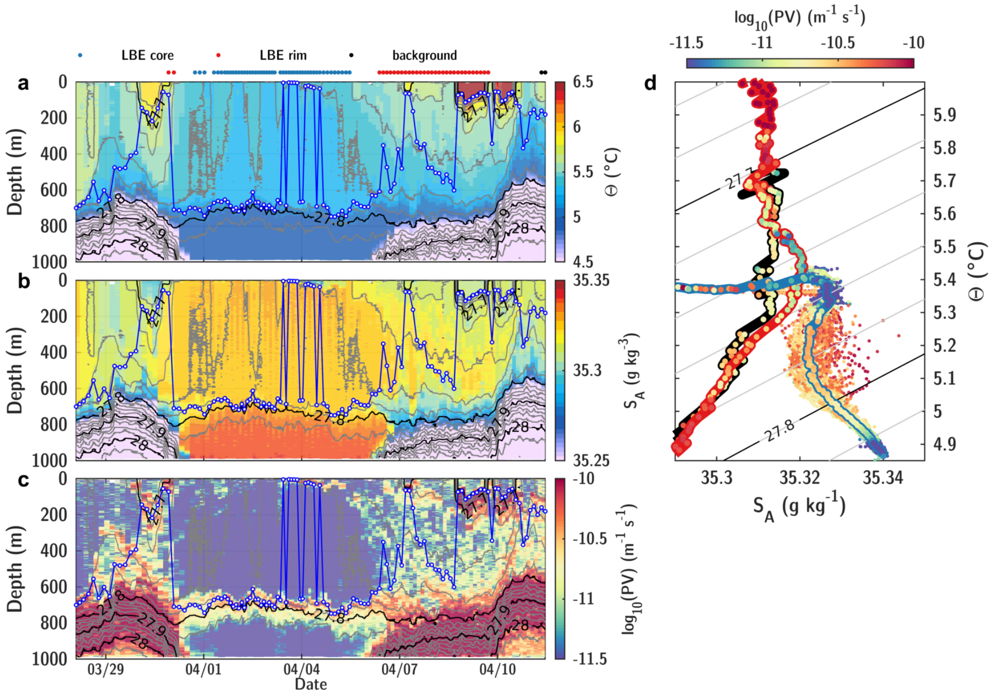

Figure S4: *Figure continuation, 2/3 : Glider sampling of the LBE in April.*

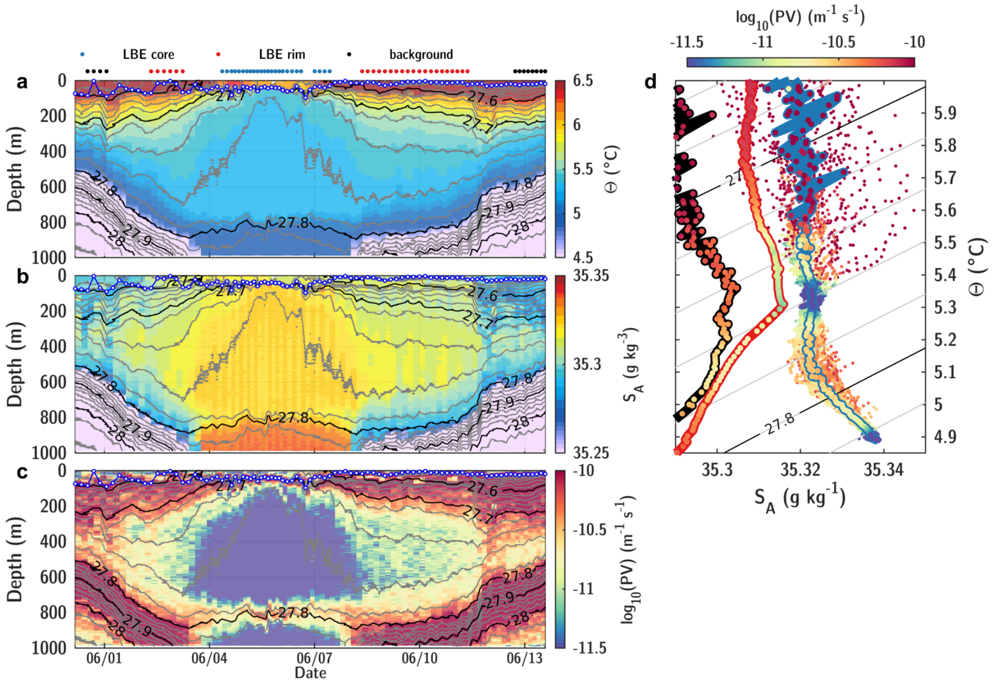

Figure S4: *Figure continuation, 3/3 : Glider sampling of the LBE in June.*

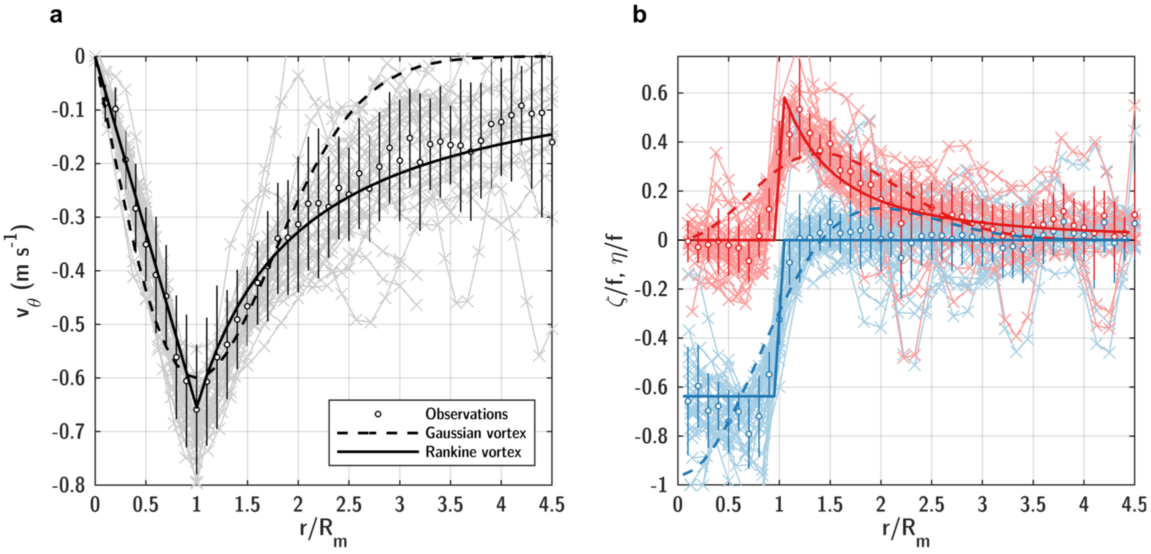

Figure S5: *(a) Orthoradial velocities as a function of distance to the eddy center normalized by the eddy radius (R_m) for each LBE radial section and average at $\pm 25\,m$ around the depth of the velocity maximum. The white dots show the bin-average values with vertical bars showing $\pm$ one standard deviation. The dashed line is the least-square fit to a Gaussian vortex ($v_\theta = v_f \frac{r}{R_m} e^{0.5[1-(r/R_m)^2]}$, with $v_f = 0.6\,m\,s^{-1}$) and the continuous line is the least-square fit to a Rankine vortex ($v_\theta(r) = v_f \frac{r}{R_m}$ for $\frac{r}{R_m} < 1$ and $v_\theta = v_f \frac{R_m}{r}$ otherwise, with $v_f = -0.65\,m\,s^{-1}$). (b) Same but for vorticity and strain rate. The dashed and continuous lines result from the least-square fitting to theoretical vortex models.*

cember. In the Θ/S_A space, the rim and core regions had similar properties in February, while in April it evolved toward different characteristics. After spring restratification, the LBE core had different T-S characteristics along isopycnals compared to the rim and background regions. This illustrates the production of different water masses by winter mixing in the core/rim/background water, and the reset of lateral mixing barriers in the LBE core after the water column has restratified.

6. Considerations on velocity, vorticity and strain rate

The orthoradial velocity, vorticity and strain rate are represented as a function of the radial distance normalized by the eddy radius for the 27 radial sections and within 50 m around the depth of velocity maximum (Figure S5). Least-square fits to the classical Gaussian and Rankine vortices were performed. The solid-body rotation core and slow

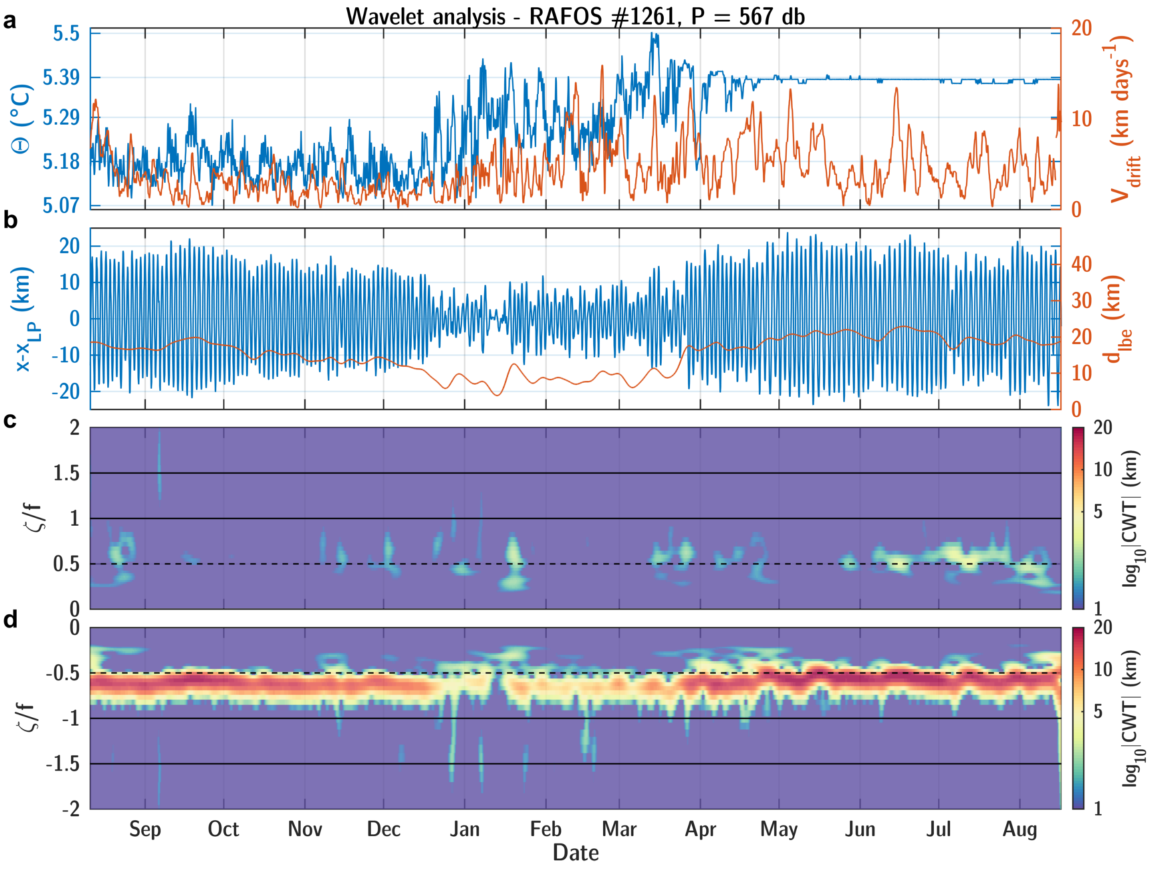

Figure S6: *(a)Temperature in blue measured by RAFOS floats #1261. LBE drift velocity in red. (b) Deviation from low-pass filtered trajectory (3rd order lowpass Butterworth filter with cutoff frequency corresponding to $0.2f$). (c-d) Signal strength in kilometers of the complex wavelet transform with counter-clockwise and clockwise components.*

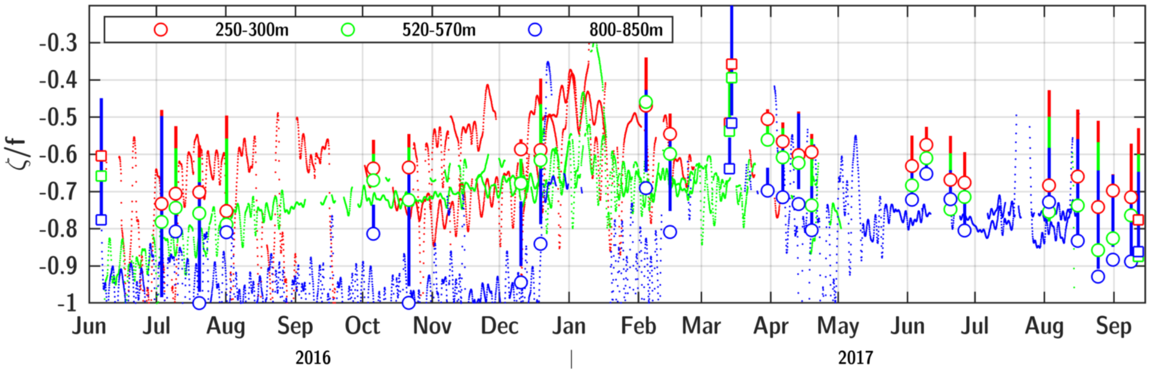

Figure S7: *Vorticity time series from RAFOS floats trapped in the LBE at different depths and within the LBE core (<10 km). Dots (resp. squares) correspond to minimal values extracted at the same depth and radial distance from glider (resp. ship) radial sections of the LBE (vertical bars show mean±standard deviation).*

decrease of velocities in the LBE agree much better with the Rankine than the Gaussian vortex model. The slow decrease in velocities can be due to interactions of the LBE with its environment [Carton, 2001].

The vorticity in the core was approximately constant, as earlier shown by Søiland and Rossby [2013] with an annulus of small positive vorticity outside of the velocity maximum (i.e., weakly shielded vortex [Carton, 2001]). The strain rate sharply increased from zero to 0.4–0.5f at about 1.2 eddy radius (i.e., about 18 km). The high strain rate region did not extend further than about 2 radius (i.e., about 30 km).

As an example, the wavelet transform of an individual RAFOS float exhibits isolated signal at scale O(1-5 km) and high Rossby number (< -1) of anticyclonic rotations from December to March (Fig. S6). The float was then trapped in the eddy core ($d_{LBE} \sim 10$ km) and the temperature increased, as a result of lateral exchanges (or vertical displacement of the eddy core).

The general trends in vorticity from Lagrangian and ship/glider estimates both agree, although they were measured differently and at different radial distance/depth. Relative vorticity in the deepest level approached $-f$ until mid-December. During winter, the ridge analysis is less successful in detecting stationary oscillatory signals, which can be an indication of the presence of more perturbations (like energetic submesoscale features). After winter, values stabilized at a weaker vorticity level. Lower vorticity was

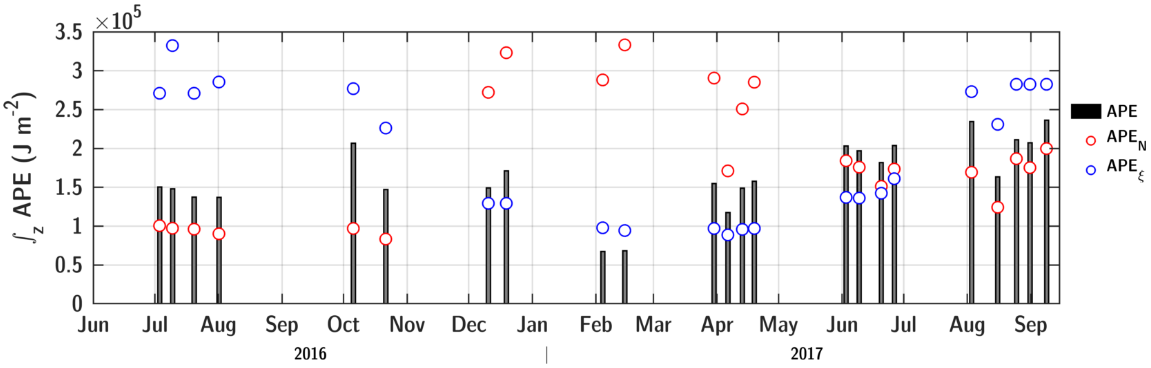

Figure S8: *Temporal variation of APE (black bars). The effects of isopycnal displacement (red) and stratification (blue) on APE is quantified by considering a fixed stratification and isopycnal displacement (average over all realization) in the integral expression of APE.*

also observed in December-February period compared to June-November, possibly due to a merger event and the lateral mixing of vorticity during the interaction.

7. Evolution of Potential Energy

In order to distinguish the influence of stratification and isopycnal displacement in APE evolution, we calculated APE_ξ and APE_N by considering respectively a fixed stratification $\bar{N}_{out}$ and isopycnal displacement $\bar{\xi}$ taken as the average over all sections (Figure S8): $APE_N(t) = \int_V 0.5\rho\bar{N}^2_{out}(z,t)\xi(r,z,t)^2 dV$ and $APE_\xi(t) = \int_V 0.5\rho N^2_{out}(z)\bar{\xi}(r,z,t)^2 dV$. APE_ξ rapidly reached a maximum when the mixing deepens in the LBE core (December to April), while APE_N varied with opposite sign by a similar order of magnitude from October to August. The early destratification (resp. late restratificaton) of the background caused the observed drop (resp. increase) in APE during the Fall-Winter (resp. Spring-Summer) transition. The difference between the LBE core and background in terms of timing and depth of the winter mixing thus governed the APE evolution during the study period. Interestingly, APE levels differ in Summer 2016 and 2017, illustrating the interannual variability in the LBE energy. The difference in APE_ξ illustrates the different states of background stratification, which are influenced by the Atlantic Water inflow (e.g., the recent AW freshening Mork et al. [2019]), as well as offshore freshwater transport across the shelf break. During winter, the low-stratified upper core expanded by winter mixing, thus increasing the isopycnal displacement observed during the follow-

ing summer. The different values of APE_N between the two summers thus reflect how the isopycnal displacements due to the LBE can be modified by wintertime convection.

References

Anthony Bosse, Ilker Fer, Henrik Søiland, and Thomas Rossby. Atlantic water transformation along its poleward pathway across the nordic seas. *Journal of Geophysical Research: Oceans*, 123(9):6428–6448, 2018. doi: 10.1029/2018JC014147.

Xavier Carton. Hydrodynamical modeling of oceanic vortices. *Surveys in Geophysics*, 22 (3):179–263, 2001. doi: 10.1023/A:1013779219578.

Kjell Arne Mork, Øystein Skagseth, and Henrik Søiland. Recent warming and freshening of the norwegian sea observed by argo data. *Journal of Climate*, 32(12):3695–3705, 2019. doi: 10.1175/JCLI-D-18-0591.1.

H. Søiland and T. Rossby. On the structure of the Lofoten Basin Eddy. *J. Geophys. Res.*, 118(9):4201–4212, 2013. doi: 10.1002/jgrc.20301.
